# NF-*κ*B-Regulated miR-99a Modulates Endothelial Cell Inflammation

**DOI:** 10.1155/2016/5308170

**Published:** 2016-06-14

**Authors:** Mei-hua Bao, Jian-Ming Li, Huai-qing Luo, Liang Tang, Qiao-li Lv, Guang-yi Li, Hong-hao Zhou

**Affiliations:** ^1^Department of Anatomy, Histology and Embryology, Institute of Neuroscience, Changsha Medical University, Changsha 410219, China; ^2^Department of Clinical Pharmacology, Xiangya Hospital, Central South University, Changsha 410008, China; ^3^Institute of Clinical Pharmacology, Central South University, Hunan Key Laboratory of Pharmacogenetics, Changsha 410078, China

## Abstract

*Objective*. The present study was performed to investigate the effects and mechanisms of miR-99a on LPS-induced endothelial cell inflammation, as well as the regulation of NF-*κ*B on miR-99a production.* Methods and Results*. ELISA showed that LPS treatment significantly promoted the secretion of inflammatory factors (TNF-*α*, IL-6, IL-1*β*, and MCP-1). LPS treatment also inhibited miR-99a production and promoted mTOR expression and NF-*κ*B nuclear translocation. Overexpression of miR-99a suppressed the LPS-induced TNF-*α*, IL-6, IL-1*β*, and MCP-1 overproduction, mTOR upregulation, and NF-*κ*B nuclear translocation. The PROMO software analysis indicated NF-*κ*B binding site in the −1643 to −1652 region of miR-99a promoter. Dual luciferase reporter analysis, electrophoretic mobility shift assays (EMSA), and chromosome immunoprecipitation (ChIP) assays demonstrated that NF-*κ*B promoted the transcription of miR-99a by binding to the −1643 to −1652 region of miR-99a promoter. Further studies on HUVECs verified the regulatory effects of NF-*κ*B on miR-99a production.* Conclusion*. MiR-99a inhibited the LPS-induced HUVECs inflammation via inhibition of the mTOR/NF-*κ*B signal. NF-*κ*B promoted miR-99a production by binding to the −1643 to −1652 region of miR-99a promoter. Considering the importance of endothelial inflammation on cardiovascular diseases, such as atherosclerosis, our results may provide a new insight into the pathogenesis and therapy of atherosclerosis.

## 1. Introduction

Atherosclerosis is a chronic inflammatory disease, which causes clinical manifestations such as ischemic stroke and coronary artery diseases. It has been a huge burden on the society. In the pathogenesis of atherosclerosis, endothelial dysfunction and inflammation have been postulated to be the initial step [1]. Approaches to suppressing the inflammation or protecting endothelial cells are important strategies for the atherosclerosis therapy [2].

MicroRNAs are noncoding, small RNAs with a length of about 22 nt. They regulate the expression of many genes at a posttranscriptional level by binding to the 3′-UTR of target genes. MiR-99a is located in chromosome 21q21.1. Recent studies have found that miR-99a contributes to cancers, focal cerebral ischemic-reperfusion injury, and T-cell differentiation by directly targeting mammalian target of rapamycin (mTOR) [3–5]. However, whether or not miR-99a interfere with endothelial functions is still unknown. mTOR is a serine/threonine kinase which plays critical roles in cell growth, proliferation, and apoptosis [6]. Recently, interactions between mTOR signal and NF-*κ*B pathway have been recognized. An Akt/mTOR/IKK*β*/NF-*κ*B signaling cascade has been reported in lung epithelial cells [7]. Since NF-*κ*B is a proinflammatory transcription factor, we assume that miR-99a might affect the endothelial inflammation though mTOR/NF-*κ*B signal.

NF-*κ*B is a multifunctional nuclear factor which regulates the transcription of several microRNAs, such as miR-224, miR-34a, let-7a-3, and miR-155 [8–10]. We also found in our pilot experiments that pyrrolidinedithiocarbamic (PDTC), an inhibitor of NF-*κ*B activation, inhibited the production of miR-99a significantly in human umbilical vein endothelial cells (HUVECs). Therefore, we hypothesize that NF-*κ*B might contribute to the production of miR-99a.

The present study was performed to investigate the effects and mechanisms of miR-99a on lipopolysaccharide- (LPS-) induced endothelial cell inflammation and to investigate the regulatory effects of NF-*κ*B on miR-99a production.

## 2. Materials and Methods

### 2.1. Materials

HEK293 and HUVECs were purchased from the American Type Culture Collection (Manassas, USA); Dulbecco's modified Eagle's medium (DMEM) and Fetal Bovine Serum were purchased from Hyclone (Utah, USA); PDTC was obtained from Sigma (MO, USA); SYBR Green qPCR SuperMix was provided by Invitrogen (California, USA); the first strand cDNA synthesis kit was purchased from Fermentas (Ottawa, Canada); the microRNA primers were provided by RIBO (Guangzhou, China); Secrete-Pair*™* Dual Luminescence Assay Kit was provided by GeneCopoeia Inc. (Rockville, USA); mouse anti-NF-*κ*B (p65) polyclonal antibody (sc-372), rabbit anti-NF-*κ*B (p50) polyclonal antibody (SC-7178), rabbit anti-mTOR polyclonal antibody (sc-136269), and the second antibody were provided by Santa Cruz Biotechnology Inc. (Texas, USA). ChIP-IT® express kit was provided by Active Motif (California, USA). TNF-*α*, IL-6, IL-1*β*, and MCP-1 ELISA kits were provided by BOSTER (Wuhan, China).

### 2.2. Cell Culture, Treatment, and Transfection

HEK293 cells were maintained in DMEM (high glucose) with 10% FBS and HUVECs were cultured in DMEM (low glucose) with 10% FBS without antibodies. Both were maintained in a humidified atmosphere containing 5% CO_2_ at 37°C. The cells between passages 2 and 15 were used in this study.

Lipofectamine 2000 (Invitrogen) was used to transfect miR-99a mimic or inhibitor, promoter plasmids, or NF-*κ*B (p50 or/and p65) plasmids. The lipofectamine 2000 or plasmids DNA were diluted in Opti-MEM I Medium (Invitrogen) and incubated for 5 min. After the incubation, transfection complexes were obtained by mixing the diluted lipofectamine 2000 and the diluted plasmids DNA and incubating for another 20 min. These transfection complexes were then added to cells and incubated for 6 hours. Subsequently, the medium was replaced by fresh normal growth medium.

### 2.3. Quantitative PCR Detection of miR-99a

Total cellular RNA was extracted using Trizol reagent according to the manufacturer's instructions. For miR-99a detection, All-in-One*™* miRNA qRT-PCR Detection Kit was used (GeneCopoeia) following the manufacturer's protocol. U6 was used as positive control. The quantitative PCR was performed using LightCycler 480 and SYBR Green system (Roche Diagnostic Systems, Somerville, NJ, USA) according to the manufacturer's protocol.

### 2.4. Enzyme-Linked Immunosorbent Assay (ELISA) of TNF-*α*, IL-6, IL-1*β*, and MCP-1

HUVECs were transfected with miR-99a mimic (100 nM) or inhibitor (150 nM) as described in Section 2.2 for 24 hours and then treated with 20 *μ*g/mL LPS for another 24 hours. The inflammatory cytokines on the supernatant were determined by ELISA methods according to manufacturer's instructions.

### 2.5. Western-Blot Analysis for NF-*κ*B (p65 and p50), *β*-actin, Histone H1, and mTOR Expression

Total cell protein was obtained by incubating cells in ice-cold RIPA buffer (containing 5 mM phenyl methyl sulfonyl fluoride (PMSF), 10 *μ*g/mL leupeptin, 1 *μ*g/mL aprotinin, and 1% NP-40). The nuclear and cytoplasmic proteins were separated, respectively, by hypotonic and hypertonic buffer as previously reported [11]. The protein concentration of cell lysates was determined by the BCA method. Cell lysates from each experiment (40 *μ*g per lane) were separated by SDS-PAGE at different concentrations and then transferred to PVDF membranes. After incubation in the blocking solution (4% nonfat milk), the membranes were incubated with a 1 : 200 dilution primary antibody for NF-*κ*B (p65 or p50), *β*-actin, Histone H1, and mTOR for overnight at 4°C. Membranes were washed and then incubated with a 1 : 10000 dilution of second antibody for 1 h, and the membranes were detected with the enhanced chemiluminescence system. Relative intensities of protein bands were analyzed by the Quantity One® software.

### 2.6. Computational Analysis of NF-*κ*B Binding Site in miR-99a Promoter

The location of miR-99a in genome was analyzed by PUBMED. The DNA sequence and the NF-*κ*B binding sites of the miR-99a promoter were analyzed and identified by the software PROMO, which can be accessed at http://alggen.lsi.upc.es/cgi-bin/promo_v3/promo/promoinit.cgi?dirDB=TF_8.3 [9, 12].

### 2.7. MiR-99a Promoter Reporter Plasmids Construction, Transfection, and Dual Luciferase Reporter Analysis

We amplified the −2.0 kb and −1.5 kb regions of miR-99a promoter using the following primers: for −1.5 kb, forward 5′-AGT TAC TTA AGC TCG GGC CC-3′, reverse 5′-TTG TTC TCG GTG GGC TTG GC-3′; for −2.0 kb, forward 5′-AGT TAC TTA ACG TCG GGC CC-3′, reverse 5′-TTG TTC TCG GTG GGC TTG GC-3′. The PCR products were cloned into the pEZX-PG04 vector (GeneCopoeia, USA) using BgIII/EcoRI and HindIII restriction enzymes to generate pEZX-PG04-2.0 Kb and pEZX-PG04-1.5 Kb plasmids. The predicted NF-*κ*B binding site TAGGGAAAAAC (map positions −1643 to −1652) was mutated to TA **TTT** AAA **GG** C using QuickChanges Kit (Stratagene, USA) with pEZX-PG04-2.0 Kb as the template and was named pEZX-PG04-2.0 Kb-Mut. The p50 ORF cDNA Clones (F0208) and p65 ORF cDNA Clones (Z4808) were purchased from GeneCopoeia (MD, USA).

For the transfection, HEK293 cells were cultured into a 24-well plate at a density of 5 × 10^4^ cells/well for 24 hours. After that, the cells were transfected with 300 ng of promoter reporter plasmids (pEZX-PG04-2.0 Kb, pEZX-PG04-1.5 Kb, pEZX-PG04-2.0 Kb mutant, or blank plasmid pEZX-PG04) with 150 ng of NF-*κ*B subunit ORF cDNA Clones (p50, p65, and p50 plus p65 (150 ng for each) or blank pReceiver-M13 vector (NC)) for 48 hours, and the luciferase activity was measured by the Secrete-Pair Dual Luciferase Assay Kit according to the manufacturer's instructions (GeneCopoeia). The secreted alkaline phosphatase was used as internal control and the results were presented as the relative ratio of Gaussia Luciferase to secreted alkaline phosphatase.

### 2.8. Electrophoretic Mobility Shift Assays (EMSA)

The Thermo Scientific LightShift Chemiluminescent EMSA Kit (Thermo Scientific Pierce) was used to detect the interactions of NF-*κ*B and miR-99a promoter. A 20 bp of probe covering the miR-99a promoter sequence between −1639 and −1658 was used for EMSA. The sequence of the wild-type probe is 5′-TTT TAG GGA AAA ACT TAA AA-3′, and the sequence of mutant-type probe is 5′-TTT TA ***T TT*** A AA ***G G*** CC TAA AA-3′ (mutations are underlined and italic). A positive NF-*κ*B probe with the sequence of 5′-AGT TGA GGG GAC TTT CCC AGG C-3′ was used. The above three DNA fragments were biotin-labeled using biotin 3′ end DNA labeling kit (Viagene Biotech Inc.). The binding reaction was performed by adding 4 *μ*g of nuclear extracts, 20 fmol of biotin end-labeled DNA (or unlabeled DNA for competitive assay), and 1 *μ*g/*μ*L poly(dI-dC) to the end volume of 15 *μ*L. After incubation for 20 min at room temperature, the reaction is then subjected to gel electrophoresis on a 6% native polyacrylamide gel and transferred to a nylon membrane. The biotin end-labeled DNA was detected using the Streptavidin-Horseradish Peroxidase Conjugate and the Chemiluminescent Substrate.

### 2.9. Chromatin Immunoprecipitation Assay (ChIP)

To further verify the binding of NF-*κ*B on endogenous miR-99a promoter, ChIP assay was performed. The HUVECs were cultured in the 6 cm plate at a density of 5 × 10^6^ cells/plate. The cells were then transfected with NF-*κ*B subunit p65 (8 *μ*g) plus p50 (8 *μ*g) as described in Section 2.2. After 48 hours of transfection, the ChIP assay was performed using the ChIP-IT express kit (Active Motif, Carlsbad, CA, USA) according to the manufacturer's instruction [13]. Briefly, cells were washed with PBS and then cross-linked with 1.5% formaldehyde at 37°C for 10 min. After washing with ice-cold PBS, cells were collected and suspended in ice-cold lysis buffer for 30 min on ice. Cell lysates were centrifuged for 10 min to pellet the nuclei. Then the nuclei pellet was sonicated using a Sonicator 3000. After that the samples were centrifuged and the supernatant containing the sheared chromatin was collected. These chromatins were used as input control. For the ChIP reaction, 25 *μ*L protein G magnetic beads, 10 *μ*L ChIP buffer, 20 *μ*g sheared chromatin, 1 *μ*L protease inhibitor cocktail, ddH_2_O, and 3 *μ*g of mouse anti-NF-*κ*B (p65) polyclonal antibody (or normal mouse IgG) were added into a total volume of 100 *μ*L. The ChIP reaction solution was then incubated on an end-to-end rotator for overnight at 4°C. The magnetic beads were pelleted on the tube side and the supernatant was discarded. After being washed with ChIP buffer, the chromatin was eluted and reverse cross-linked with proteinase K. The DNA was used immediately in PCR. For the PCR, 1.5 *μ*L of the DNA was amplified with Taq DNA polymerase, using a primer pair specific to the binding region of miR-99a promoter (forward primer, 5′-CGT CTA CCC TCA TTC CCA CG-3′; reverse primer: 5′-TGG GAC ACA AAC TGC CCA AT-3′, 36 cycles, 353 bp). The annealing temperatures were 57.3°C. Finally, the PCR amplified samples were visualized on 1.5% agarose gels using Quantity One for analysis.

### 2.10. Statistical Analysis

All data were presented in the statistics of three independent experiments in the form of mean ± SD. The significance of the difference was analyzed by ANOVA followed by Newman-Student-Keuls test. A value of *P* < 0.05 was considered statistically significant.

## 3. Results

### 3.1. Effects of miR-99a on LPS-Induced Endothelial Cell Inflammation

**Figure 1:**
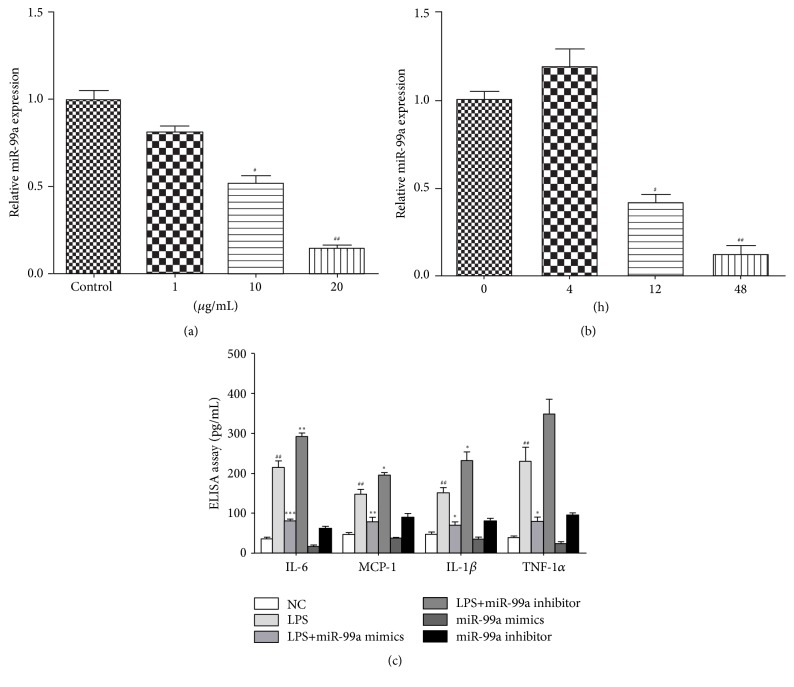
Effects of LPS on miR-99a production and effects of miR-99a on LPS-induced inflammation in HUVECs. (a) Effects of LPS treatment at different concentrations for 24 hours on miR-99a expression; (b) effects of different time treatment with LPS (20 *μ*g/mL) on miR-99a expression; (c) effects of miR-99a on LPS-induced inflammatory factors secretion. The values (mean ± SD from three independent experiments). ^#^ *P* < 0.05; ^##^ *P* < 0.01 versus control; ^*∗*^ *P* < 0.05; ^*∗∗*^ *P* < 0.01 versus LPS group; ^*∗∗∗*^ *P* < 0.001 versus LPS group.

As shown in Figures 1(a) and 1(b), LPS treatment inhibited the expression of miR-99a in a concentration- and time-dependent manner. High concentrations of LPS (10 and 20 *μ*g/mL) significantly inhibited the expressions of miR-99a. 12 and 24 hours of LPS (20 *μ*g/mL) treatment also inhibited the level of miR-99a significantly.

To explore the role of miR-99a on LPS-induced endothelial inflammation, we detected the expression of inflammatory factors by ELISA. As shown in Figure 1(c), LPS treatment induced IL-6, MCP-1, IL-1*β*, and TNF-1*α* expression compared with the control group, and this overexpression in HUVECs could be inhibited by miR-99a significantly. On the other hand, miR-99a inhibition elevated the LPS-induced IL-6, MCP-1, IL-1*β*, and TNF-1*α* expression. Moreover, miR-99a alone also inhibited the inflammatory factors secretion slightly.

### 3.2. Effects of miR-99a on mTOR/NF-*κ*B Pathway

**Figure 2:**
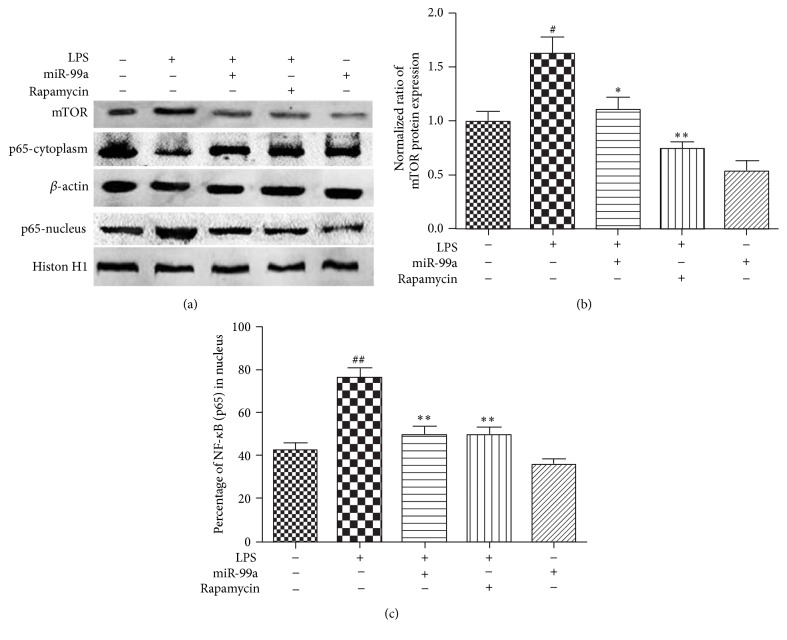
Effects of miR-99a on LPS-induced mTOR expression and NF-*κ*B nuclear translocation. Endothelial cells were transfected with miR-99a (100 nM) mimic or rapamycin (50 nM) for 24 hours and then treated with LPS (20 *μ*g/mL) for another 24 hours. The protein levels were detected by Western-blot. (a) The expression of different proteins analyzed by Western-blot; (b) quantified band density for mTOR relative to control, which was set as 1; (c) percentage of NF-*κ*B in nucleus, total NF-*κ*B presented by the summary of quantified band density for NF-*κ*B (cytoplasm) and NF-*κ*B (nucleus). All values are presented as mean ± SD from three independent experiments. ^#^ *P* < 0.05; ^##^ *P* < 0.01 versus control; ^*∗*^ *P* < 0.05; ^*∗∗*^ *P* < 0.01 versus LPS.

As shown in Figure 2, LPS (20 *μ*g/mL) treatment induced the mTOR expression and NF-*κ*B nuclear translocation significantly. MiR-99a overexpression suppressed the LPS-induced mTOR upregulation and NF-*κ*B nuclear translocation. We also found that rapamycin suppressed mTOR expression, as well as NF-*κ*B nuclear translocation induced by LPS. And miR-99a overexpression alone inhibited the mTOR protein expression.

### 3.3. Promotion Effects of NF-*κ*B on miR-99a Expression in HUVECs

**Figure 3:**
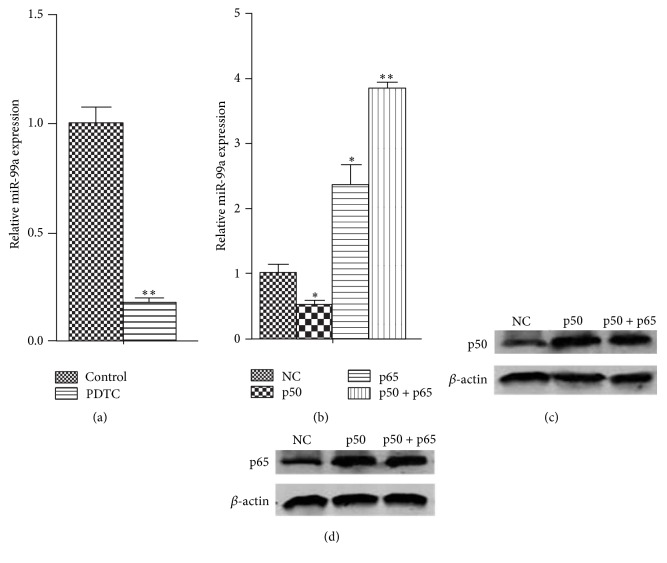
Regulatory effects of NF-*κ*B on miR-99a expression in HUVECs. ((a), (b)) Relative miR-99a expression in HUVECs after NF-*κ*B overexpression or inhibition. ((c), (d)) The increased p50 or p65 protein levels in HUVECs which were transfected with 4 *μ*g of NF-*κ*B subunit ORF cDNA Clones (p50, p65, or p50 + p65) in 6-well plate for 48 hours. The values (mean ± SD from three independent experiments) are relative to NC or control, which was set as 1. ^*∗*^ *P* < 0.05; ^*∗∗*^ *P* < 0.01 versus NC group.

In order to detect the regulatory effects of NF-*κ*B on miR-99a expression, we measured the miR-99a expression in HUVECs after NF-*κ*B overexpression or inhibition by real-time PCR. As shown in Figure 3, after 48 hours of transfection, the p50 and p65 expression increased dramatically (Figures 3(c) and 3(d)). It can be observed that p65 overexpression and p50 + p65 overexpression promoted the miR-99a expression (Figure 3(b)). Interestingly, the results showed that p50 overexpression exhibited inhibition effects on miR-99a expression. Since p50 overexpression can cause p50 dimer formation, this might be due to the blocking of promoter region of miR-99a by p50 dimer. Furthermore, after the treatment with PDTC, an inhibitor of the NF-*κ*B activation, the miR-99a levels were decreased dramatically, which confirmed the promoting effects of NF-*κ*B on miR-99a expression (Figure 3(a)).

### 3.4. Regulatory Effects of NF-*κ*B on miR-99a Promoter Plasmids

**Figure 4:**
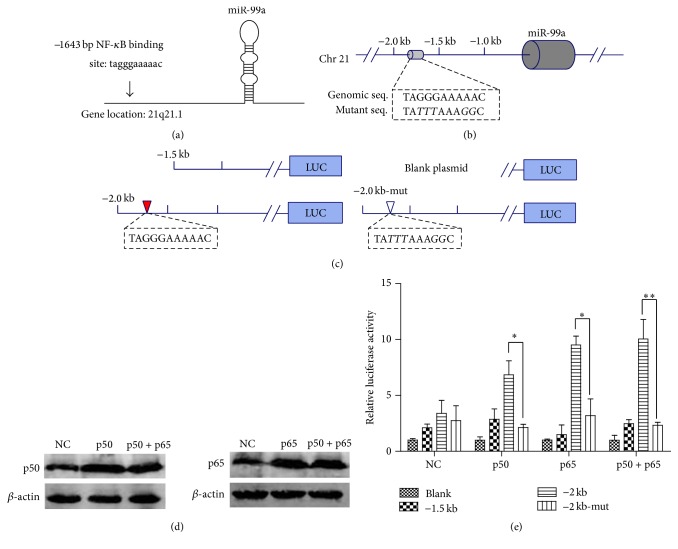
Dual luciferase reporter analysis of the regulate effects of NF-*κ*B on miR-99a promoter plasmids. (a) The predicted NF-*κ*B binding sites in miR-99a promoter analyzed by PROMO software. ((b), (c)) Portions of the upstream region of miR-99a cloned to create −1.5 kb, −2.0 kb, and −2.0 kb-Mut plasmids. (d) The increased p65 or p50 protein levels in HEK293 cells after being transfected with 150 ng of NF-*κ*B subunit ORF cDNA Clones (p50, p65, or p50 + p65) in 24-well plates for 48 hours. (e) Relative luciferase activity of different promoter reporter plasmids after NF-*κ*B overexpression. The values (mean ± SD from three independent experiments) are relative to NC-Blank, which was set as 1. ^*∗*^ *P* < 0.05; ^*∗∗*^ *P* < 0.01.

PROMO software analysis discovered NF-*κ*B binding site at −1643 bp to −1652 bp region of the miR-99a promoter, which are coincident with a previous report, which indicates a potential promoter region in the −1.0 kb to −2.0 kb of miR-99a [14] (Figures 4(a) and 4(b)). Therefore, we cloned −1.5 kb and −2.0 kb of miR-99a upstream regions into the pEZX-PG04 vectors (Figure 4(c)). The vectors were transfected into HEK293 cells accompanied with different NF-*κ*B subunit (p50, p65, or p50 + p65). After 48 hours of transfection, the p50 or p65 expression increased dramatically (Figure 4(d)). As shown in Figure 4(e), the NF-*κ*B subunits (p50, p65, or p50 + p65) overexpression significantly induced the luciferase activities of pEZX-PG04-2.0 Kb plasmids. However, they have no effects on the luciferase activities of pEZX-PG04-1.5 Kb plasmids and pEZX-PG04-2.0 Kb-Mut plasmids (*P* > 0.05, compared with NC). Compared with pEZX-PG04-2.0 Kb-Mut group, the luciferase activities were dramatically higher in pEZX-PG04-2.0 Kb group after p50 and p65 overexpressions.

### 3.5. EMSA and ChIP Validation of NF-*κ*B Binding Sites on miR-99a Promoter

**Figure 5:**
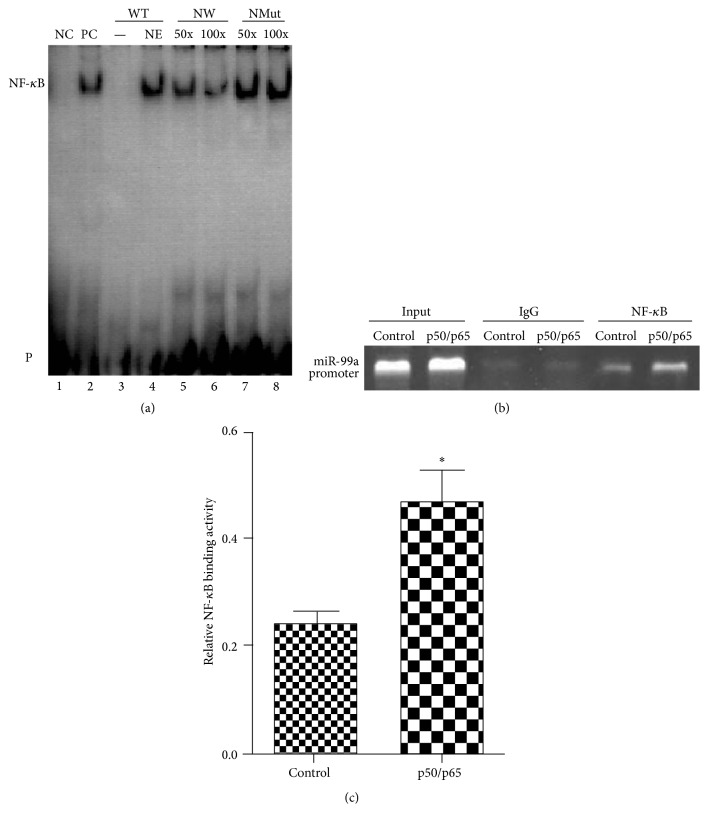
EMSA and ChIP verification of interactions of NF-*κ*B with its binding sites in the miR-99a promoter. (a) EMSA validation of NF-*κ*B binding sites on miR-99a promoter. Lane 1, labeled positive NF-*κ*B probe only; lane 2, nuclear extracts plus labeled positive NF-*κ*B probe; lane 3, labeled WT probe only; lane 4, nuclear extracts plus labeled WT probe; lanes 5 and 6, nuclear extracts plus labeled WT probe and cold unlabeled WT probe (50-fold and 100-fold labeled WT probe), respectively; lanes 7 and 8, nuclear extracts plus labeled WT probe and cold unlabeled Mut probe (50-fold and 100-fold labeled WT probe), respectively. NC: negative control; PC: positive control; NE: nuclear extracts; WT: wild-type probe; NWT: nonlabeled wild-type probe; NMut: nonlabeled mutant probe; P: free biotin-labeled probe. (b) Representative gel images of ChIP assay. (c) Relative NF-*κ*B binding activity quantified by the ChIP bands. ^*∗*^ *P* < 0.05 versus control group.

For the EMSA, double-strand synthetic oligonucleotides encompassing the region from −1639 bp to −1658 bp were biotin labeled and incubated with NF-*κ*B positive control protein and then analyzed by nondenaturing polyacrylamide gel electrophoresis. As shown in Figure 5, the reaction of biotin-labeled WT probe with nuclear extracts led to the emergence of a shifted DNA-protein complex (Figure 5, lane 4), which was coincident with the shift band for positive NF-*κ*B probe (Figure 5, lane 2, PC). The WT probe and nuclear extracts binding were specifically competed off by the addition of a 50-fold or 100-fold excess of unlabeled WT probe (Figure 5(a), lane 5 and lane 6), but not by the unlabeled Mut probe (Figure 5(a), lane 7 and lane 8).

To further confirm the binding of NF-*κ*B on HUVEC endogenous miR-99a promoter, ChIP assay was performed. The assay showed that NF-*κ*B (p65 plus p50) overexpression for 48 hours significantly increased the binding of NF-*κ*B to the miR-99a promoter in HUVEC (Figures 5(b) and 5(c)).

## 4. Discussion

In the present study, we investigated the effects and mechanisms of miR-99a on LPS-induced HUVECs inflammation, as well as the regulatory effects of NF-*κ*B on miR-99a production. Our results demonstrated that overexpression of miR-99a inhibited the LPS-induced endothelial cell inflammation. The mTOR/NF-*κ*B signal might be involved in the effects of miR-99a. NF-*κ*B promoted the transcription of miR-99a by binding to the −1643 to −1652 region of miR-99a promoter.

MiR-99a is a microRNA associated with diseases such as cancers, focal cerebral ischemia-reperfusion injury by interfering the processes of cell proliferation, apoptosis, and inflammation [3, 15, 16]. One of the targets of miR-99a is reported to be mTOR [15, 17]. Our present study found that LPS, a widely known proinflammation factor, inhibited the expression of miR-99a. Overexpression of miR-99a suppressed the LPS-induced inflammatory cytokines secretion. These findings suggest the anti-inflammatory effects of miR-99a (Figure 1).

LPS has been demonstrated to promote the inflammatory response through NF-*κ*B [18]. A very recent study shows that LPS stimulates the NF-*κ*B signal and cytokines secretion partly due to mTOR activation [19]. Recently, the relationships between mTOR and NF-*κ*B have been identified. Hou et al. demonstrated the Akt/mTOR/IKK*β*/NF-*κ*B signaling cascade in lung epithelial cells [20]. Lin et al. found that sirolimus, an inhibitor of mTOR, significantly suppressed the LPS-induced inflammatory in monocytes via the inhibition of p38 MAPK and NF-*κ*B signaling pathways [7]. These studies indicate that mTOR might be the upstream event of NF-*κ*B signaling. Since miR-99a interferes with the mTOR expression, we presume that the anti-inflammatory effects of miR-99a are obtained through mTOR/NF-*κ*B signaling. Our studies demonstrated a significant decrease of NF-*κ*B activation after mTOR suppression in HUVECs, suggesting the existence of mTOR/NF-*κ*B in this cell type. When miR-99a is overexpressed, the mTOR level and NF-*κ*B activation are decreased, indicating that miR-99a is involved in the mTOR/NF-*κ*B signaling (Figure 2).

NF-*κ*B is a multifunctional nuclear factor which regulates the transcription of several miRNAs [8–10]. In the present study, we found a dramatic decrease in miR-99a level by the inhibition of NF-*κ*B activation by PDTC in HUVECs (Figure 3(a)). Therefore, we cloned the promoter region of miR-99a into reporter gene plasmids and performed analysis through dual luciferase assays. We found that NF-*κ*B overexpression promoted the luciferase activity of plasmids covering the predicted NF-*κ*B binding site (−1643 to −1652) but had no effects on binding site mutant plasmids and shortened plasmids (pEZX-PG04-1.5 Kb) (Figure 4). The binding of NF-*κ*B on miR-99a promoter (−1643 to −1652) was confirmed by EMSA and ChIP assays (Figure 5). Our results validated previous hypothesis of the existence of a putative promoter at the −1.0 to −2.0 region of mi-99a [14, 21, 22].

Surprisingly, we found that LPS, an activator of NF-*κ*B, inhibited the expression of miR-99a, which was opposite to the enhanced effects of NF-*κ*B ORF cDNA Clones on miR-99a production. These conflicting results might be explained by the more complicated response of HUVECs to LPS treatment than to the NF-*κ*B ORF cDNA Clones transfection. It was reported that LPS also induced the expression of c-Myc and hypermethylation in addition to NF-*κ*B [23–25]. For the production of miR-99a, previous studies have reported the modulation effects of HOXA1, c-Myc, hypermethylation, androgen on it [26–29]. Thus the LPS-induced increase in c-Myc or other unknown factors or the hypermethylation may inhibit miR-99a production in HUVECs. Therefore, when treated with LPS, the inhibition factors might overcome the promoting factors and eventually result in the decrease of miR-99a in HUVECs. These results, taken together with ours, suggest a complex regulatory network for miR-99a production.

In conclusion, the present studies demonstrated that, in LPS-treated endothelial cells, miR-99a inhibited the mTOR expression, suppressed the nuclear translocation of NF-*κ*B, and resulted in the attenuation of inflammation. Upregulation of NF-*κ*B promoted the production of miR-99a directly. Our results showed a novel mechanism for miR-99a generation. Considering the important roles of endothelial inflammation in atherosclerosis, our results may provide a new insight into the pathogenesis of atherosclerosis.
